# Validation of a modified Chinese version of Mini‐Addenbrooke's Cognitive Examination for detecting mild cognitive impairment

**DOI:** 10.1002/brb3.2418

**Published:** 2021-11-29

**Authors:** Feng‐Feng Pan, Liang Cui, Qing‐Jie Li, Qi‐Hao Guo

**Affiliations:** ^1^ Department of Gerontology Shanghai Jiao Tong University Affiliated Sixth People's Hospital Shanghai China

**Keywords:** Alzheimer's disease, mild cognitive impairment, Mini‐Addenbrooke's Cognitive Examination, Mini‐Mental State Examination, Montreal Cognitive Assessment

## Abstract

**Background:**

For detecting mild cognitive impairment (MCI), brief cognitive screening tools are increasingly required for the advantage of time saving and no need for special equipment or trained raters. We aimed to develop a modified Chinese version of Mini‐Addenbrooke's Cognitive Examination (C‐MACE) and further evaluate its validation in detecting MCI.

**Methods:**

A total of 716 individuals aged from 50 to 90 years old were recruited, including 431 cognitively normal controls (NC) and 285 individuals with MCI. The effect size of Cramer's V was used to explore which items in the Chinese version of Addenbrooke's Cognitive Examination‐III (ACE‐III‐CV) best associated with MCI and to form the C‐MACE. Receiver operating characteristic (ROC) analyses were carried out to explore the ability of C‐MACE, ACE‐III‐CV, Chinese version of Montreal Cognitive Assessment‐Basic (MoCA‐BC), and Mini‐Mental State Examination (MMSE) in discriminating MCI from NC.

**Results:**

Five items with greatest effect sizes of Cramer's V were selected from ACE‐III‐CV to form the C‐MACE: Memory Immediate Recall, Memory Delayed Recall, Memory Recognition, Verbal Fluency Animal and Language Naming. With a total score of 38, the C‐MACE had a satisfactory classification accuracy in detecting MCI (area under the ROC curve, AUC = 0.892), superior to MMSE (AUC = 0.782) and comparable to ACE‐III‐CV (AUC = 0.901) and MoCA‐BC (AUC = 0.916). In the subgroup of Age > 70 years, Education ≤ 12 years, the C‐MACE got a highest classification accuracy (AUC = 0.958) for detecting MCI.

**Conclusion:**

In the Chinese‐speaking population, C‐MACE derived from ACE‐III‐CV may identify MCI with a good classification accuracy, especially in aged people with low education.

## Graphical abstract

Five items from ACE‐III‐CV were selected to form C‐MACE, including: Immediate Recall, Delayed Recall, Recognition, Verbal Fluency and Naming. This C‐MACE had a satisfactory classification accuracy in detecting MCI than MMSE and comparable to the ACE‐III‐CV and Chinese version of MoCA‐Basic.

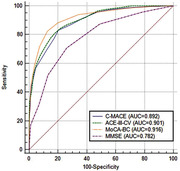

## 1 INTRODUCTION

Mild cognitive impairment (MCI) is regarded as a transitional cognitive state between normal aging and dementia (Petersen, 2016). As most dementia can hardly be reversed, it is important to identify individuals with MCI for early intervention and to decrease the risk of further cognitive decline (Langa & Levine, 2014). Up to now, neuropsychological tests still play an important role in identifying MCI. Though comprehensive neuropsychological assessments performed well in the diagnosis of MCI (Jak et al. 2009), brief cognitive assessments are consistently required for the advantage of time saving and no need for special test equipment or trained raters, especially in primary care settings and large populations. Besides the widely used cognitive screening tests of Mini‐Mental State Examination (MMSE) (Folstein et al., 1975) and Montreal Cognitive Assessment (MoCA) (Nasreddine et al., 2005), Addenbrooke's Cognitive Examination (ACE) is a designed for the detection and classification of dementia which includes subtests of orientation, attention, verbal fluency, memory, language, and visuospatial ability (Mathuranath et al., 2000). By covering the key cognitive domains, ACE showed better ability than MMSE and MoCA in predicting the development of dementia (Lischka et al., 2012). In order to increase the sensitivity and facilitate cross‐cultural usage and translation, contents in memory, language, and visuospatial domains of ACE were modified to form the revised version of ACE (ACE‐R) (Mioshi et al., 2006). By further addressing several weakness items in ACE‐R, the latest third version of ACE (ACE‐III) correlated well with the standardized neuropsychological tests and showed high diagnostic accuracy for both MCI and dementia (Bruno & Vignaga, 2019; Hsieh et al., 2013). However, as a comprehensive cognitive screening test, it takes about 15–20 min to complete the whole items of ACE‐III, a time frame that may be beyond the scope of many busy clinical settings. For addressing this shortage, five items (time orientation, 7‐element name and address immediate recall and delayed recall, animal verbal fluency, and clock drawing) derived from ACE‐III were used to develop a short version of ACE‐III named Mini‐Addenbrooke's Cognitive Examination (MACE) with a total score of 30 and takes under 5 min to administer (Hsieh et al., 2015). For discriminating individuals with MCI from normal controls (NC), previous studies showed that the reliability and classification accuracy of MACE was superior to MMSE and equivalent to MoCA to a certain extent (Larner, 2017; Senda et al., 2020; Yang et al., 2019). However, latest systematic review indicated that this published version of MACE had a variable sensitivity and more variability in specificity according to the cutoff established in index study (Beishon et al., 2019). Furthermore, due to the linguistic and cultural diversities, previous study even showed a not ideal validation of the original MACE in discriminating MCI from control subjects (Miranda et al., 2018).

So far, ACE‐R and ACE‐III have been translated into different languages and the most culturally adapted items in ACE‐III and its predecessors were anterograde memory, retrograde memory, and language (Mirza et al., 2017). Similarly, our previously published Chinese version of Addenbrooke's Cognitive Examination‐III (ACE‐III‐CV) was translated and mainly culture adapted in the items of anterograde memory, verbal fluency, phrase repetition, and visuospatial perception (Pan et al., 2021). Though this Chinese version of ACE‐III showed high ability to differentiate MCI from normal controls, the established cutoff scores and corresponding sensitivity and specificity differed significantly from other versions (Li et al., 2019; Peixoto et al., 2018). Thus, MACE derived from the original version of ACE‐III may lead to a diagnostic heterogeneity in the Chinese population. The aim of this study was to derive a modified Chinese version of Mini‐Addenbrooke's Cognitive Examination (C‐MACE) from ACE‐III‐CV and further compare the classification accuracy of this C‐MACE in detecting MCI with ACE‐III‐CV, MoCA, and MMSE.

## 2 METHODS

### 2.1 Participants

A total of 716 individuals were recruited to Shanghai Sixth People's Hospital through internet‐based and print advertisements, including 285 individuals with MCI and 431 normal controls (NC). The inclusion criteria were as follows: age between 50 and 90; education level more than 1 year; normal vision and hearing to complete cognitive tests; Clinical Dementia Rating (CDR) (Juva et al., 1995) score ≤0.5; preserved basic activities of daily living (ADL) (Chen et al., 1995); Hamilton depression rating scale (17 items) (Hamilton, 1960) score ≤12. Relevant laboratory examination and magnetic resonance imaging (MRI) scanning of the head were carried out. In addition to the history of psychiatric disorders, epilepsy, head trauma, alcoholism and substance abuse, individuals with obvious neurologic disease and significant abnormalities in folic acid, vitamin B12, thyroid function, and syphilis serology were all excluded. All the participants did not meet the diagnostic criteria of dementia recommended by the National Institute on Aging‐Alzheimer's Association (NIA‐AA) (Mckhann et al., 2011). The present study was approved by the ethics committee of Shanghai Jiao Tong University Affiliated Sixth People's Hospital. Written informed consent was obtained from all the participants.

### 2.2 Neuropsychological assessment and diagnostic criteria

Besides the cognitive screening tests of ACE‐III‐CV, MoCA, and MMSE, a battery of neuropsychological tests were carried out to diagnose MCI according to an actuarial neuropsychological method proposed by Jak and Bondi (Bondi et al., 2014). In this method, six standardized neuropsychological tests were included: Auditory Verbal Learning Test (AVLT) 30‐min delayed recall and AVLT recognition for the measurements of memory (Zhao et al., 2015); Animal Verbal Fluency Test (AFT) (Zhao et al., 2013) and 30‐item Boston Naming Test (BNT) (Guo et al., 2006) for the measurements of language; Shape Trail Test Part A and B (STT‐A, STT‐B) (Zhao et al., 2013) for the measurements of executive function. Each test has been widely used in China and was standardized using published normative data. Global functional status was also assessed by Functional Assessment Questionnaire (FAQ) (Pfeffer et al., 1982) according to informants. Participants met one of the following criteria were diagnosed as MCI: (1) impaired scores (defined as >1 standard deviation (SD) below the age‐corrected normative mean) on two of the six neuropsychological indexes in the same cognitive domain (memory, language, or executive function); (2) impaired scores (defined as >1 SD below the age‐corrected normative mean) in each of the three cognitive domains; (3) FAQ score ≥9. All the screening tests and standardized neuropsychological tests were administered on the same day by raters who were blind to the diagnosis.

### 2.3 Statistical analyses

All the items in ACE‐III‐CV were evaluated using Cramer's V, respectively, to determine which items best indicate a strong association with MCI. The effect size of Cramer's V ranges from 0 to 1 and greater than 0.5 is generally taken as a large value. The best items were supposed to have the largest effect sizes and would be selected as the items to form C‐MACE. Internal consistency of the C‐MACE was verified by the Cronbach's coefficient alpha. Spearman correlations were calculated to test the relationship between derived items and standardized neuropsychological tests, and further evaluate the convergent validity of the C‐MACE against MoCA‐BC. Independent sample *t*‐test was used to conduct the comparison of demographic characteristics and neuropsychological assessments between the group of MCI and NC, Levene's test was used to examine the equality of variance. Chi‐square test was applied to categorical data. Receiver operating characteristic (ROC) analyses were administered to explore the ability of C‐MACE, ACE‐III‐CV, MoCA‐BC, and MMSE in discriminating individuals with MCI from NC. With the method proposed by Delong et al. (1988), area under the ROC curve (AUC) was used to compare the classification accuracy of these screening tests. Sensitivity and specificity of each test according to the optimal cutoff score were determined by the maximum Youden index. Comparison of independent ROC curves was carried out to explore different classification accuracies of each screening test in different age and education level groups. The level of significance was set at *α* = 0.05. Statistical analyses were conducted using IBM SPSS Statistics 23.0 and MedCalc 19.1.

## 3 RESULTS

### 3.1 C‐MACE derived from ACE‐III‐CV

A total of 22 items in the ACE‐III‐CV were analyzed with Cramer's V to determine which ones best correlated with MCI. As a result, two items had large effect sizes (Cramer's V > 0.500, *p* < .001): 0.560 for Memory Delayed Recall; 0.510 for Verbal Fluency Animal. Three items got close to large effect sizes: 0.497 for Memory Recognition; 0.489 for Language Naming, and 0.469 for Memory Immediate Recall. The others had small to medium effect sizes no more than 0.4. These five items best indicated a strong association with MCI were selected and formed C‐MACE with a total score of 38. Compared to the version put forward by Hsieh et al. (2015), this C‐MACE contains the items of immediate recall, delayed recall, and animal verbal fluency as well, but brings in the items of recognition and naming instead of time orientation and clock drawing. The Cronbach's coefficient alpha for C‐MACE was 0.772, which indicates a high level of internal consistency. The items derived from ACE‐III‐CV were all well correlated with the standardized neuropsychological tests: immediate recall item best correlated with AVLT immediate recall (*r* = 0.423), delayed recall and recognition items best correlated with AVLT delayed recall (*r* = 0.469 and 0.410), animal verbal fluency item best corrected with AFT (*r* = 0.811), naming item best correlated with BNT (*r* = 0.721). Correlation coefficient between the C‐MACE and MoCA‐BC was 0.678 (*p* < .001), which indicated as an acceptable convergent validity. No obvious ceiling and floor effects appeared in the C‐MACE as no subject obtained a maximum total score of 38 in normal controls (28.78 ± 4.21) or a total score of zero in individuals of MCI (20.51 ± 5.08).

### 3.2 Demographics and neuropsychological tests in the group of NC and MCI

The demographics, scores of standardized neuropsychological tests, global function tests, and cognitive screening tests for the groups of NC and MCI are shown in Table 1. Compared to the normal controls, individuals with MCI were more aged and had a lower education level. Individuals with MCI performed significantly worse in the standardized neuropsychological tests (scored lower in AVLT, BNT, AFT and scored higher in STT) and got a worse functional status (scored higher in FAQ and ADL). For cognitive screening tests, scores of the C‐MACE, ACE‐III‐CV, MoCA‐BC, and MMSE were all significantly lower in the group of MCI, score gaps between MCI and NC were ranked in the order of ACE‐III‐CV > C‐MACE > MoCA‐BC > MMSE.

**TABLE 1:** Demographics and neuropsychological tests for NC and MCI

Index	NC (*n* = 431)	MCI (*n* = 285)	*t* / *χ^2^*	*p* value
***Demographics***				
Age (years)	66.52 ± 9.32	72.12 ± 10.45	−7.318	<.001
Education (years)	12.85 ± 3.32	11.67 ± 4.01	4.138	<.001
Sex (M:F)	176:255	143:142	6.058	<.001
***Standardized neuropsychological tests***				
AVLT immediate recall	18.87 ± 4.30	13.29 ± 3.61	13.704	<.001
AVLT delayed recall	6.12 ± 2.28	2.63 ± 2.12	14.642	<.001
AVLT recognition	21.95 ± 1.93	18.72 ± 2.87	11.482	<.001
BNT	24.35 ± 2.85	20.11 ± 4.35	9.989	<.001
AFT	18.29 ± 3.72	13.56 ± 3.81	10.461	<.001
STT‐A	46.26 ± 14.46	64.89 ± 31.23	−6.355	<.001
STT‐B	120.58 ± 32.98	159.88 ± 51.87	−7.728	<.001
***Global functional tests***				
FAQ	0.52 ± 1.41	2.04 ± 3.87	−5.734	<.001
ADL	20.26 ± 1.42	21.56 ±4.24	−4.519	<.001
***Cognitive screening tests***				
C‐MACE	28.78 ± 4.21	20.51 ± 5.08	22.562	<.001
ACE‐III‐CV	83.49 ± 6.47	69.46 ± 8.86	22.955	<.001
MoCA‐BC	26.27 ± 3.52	20.52 ± 3.36	21.920	<.001
MMSE	28.26 ± 1.47	26.27 ± 2.13	13.688	<.001

*Abbreviations*: ACE‐III‐CV, Chinese version of Addenbrooke's Cognitive Examination III; ADL, activities of daily living; AFT, animal verbal fluency test; AVLT, auditory verbal learning test; BNT, Boston naming test; FAQ, Functional Assessment Questionnaire; MACE, Mini‐Addenbrooke's Cognitive Examination; MCI, mild cognitive impairment; MMSE, Mini‐Mental State Examination; MoCA‐BC, Chinese version of Montreal Cognitive Assessment‐Basic; NC, normal control; STT‐A and B, Shape Trail Test Part A and B.

### 3.3 ROC analyses of C‐MACE, ACE‐III‐CV, MoCA‐BC, and MMSE for discriminating MCI from NC

**FIGURE 1:**
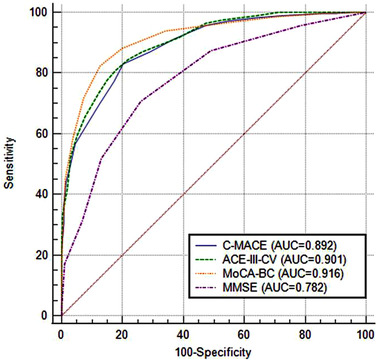
Receiver operating characteristic curves of Chinese version of Mini‐Addenbrooke's Cognitive Examination (C‐MACE), Chinese version of Addenbrooke's Cognitive Examination III (ACE‐III‐CV), Chinese version of Montreal Cognitive Assessment‐Basic (MoCA‐BC), and Mini‐Mental State Examination (MMSE) for differentiating MCI from NC

As shown in Table 2, ROC analyses were administered to evaluate the ability of C‐MACE, ACE‐III‐CV, MoCA‐BC, and MMSE in discriminating MCI from NC. For the C‐MACE derived from ACE‐III‐CV, the AUC was 0.892, with an optimal cutoff score of 25 (sensitivity 83.03% and specificity 79.81%). The AUCs for ACE‐III‐CV, MoCA‐BC, and MMSE were 0.901, 0.916, and 0.782, respectively, and the optimal cutoff scores were 77 for ACE‐III‐CV (sensitivity 81.05%, specificity 82.37%), 23 for MoCA‐BC (sensitivity 82.39%, specificity 87.47%), and 27 for MMSE (sensitivity 70.88%, specificity 74.01%). Pairwise comparison of ROC curves was displayed in Figure 1. The AUCs of the C‐MACE, ACE‐III‐CV, and MoCA‐BC all showed significantly higher than MMSE (*p* < .0001), though no significant difference was found between the C‐MACE, ACE‐III‐CV, and MoCA‐BC.

**TABLE 2:** ROC analyses for the C‐MACE, ACE‐III‐CV, MoCA‐BC, and MMSE to differentiate individuals with MCI from NC

Index	AUC	95% Confidence interval	Cutoff	Sensitivity (%)	Specificity (%)
C‐MACE	0.892	0.866–0.914	25	83.03	79.81
ACE‐III‐CV	0.901	0.877–0.922	77	81.05	82.37
MoCA‐BC	0.916	0.893–0.936	23	82.39	87.47
MMSE	0.782	0.750–0.812	27	70.88	74.01

*Abbreviations*: ACE‐III‐CV, Chinese version of Addenbrooke's Cognitive Examination III; AUC, area under the receiver operating characteristic curve; MACE, Mini‐Addenbrooke's Cognitive Examination; MCI, mild cognitive impairment; MMSE, Mini‐Mental State Examination; MoCA‐BC, Chinese version of Montreal Cognitive Assessment‐basic; NC, normal control.

### 3.4 Comparison of AUCs according to different age and education level

As the performance of screening tests was probably affected by age and education level, classification accuracies of the C‐MACE, ACE‐III‐CV, MoCA‐BC, and MMSE were further compared in different age and education level groups. Subjects in this study were divided into four subgroups according to the ages and formal education years: Age ≤ 70 years, Education ≤ 12 years; Age ≤ 70 years, Education > 12 years; Age > 70 years, Education ≤ 12 years; Age > 70 years, Education > 12 years. The education level was divided according to the current situation of Chinese education, that is, 12 years for educated from senior high school, and more than 12 years for the education in universities. The age cutoff of 70 years was chosen as dementia rises steeply in persons aged more than 70 years (Bachman et al., 1993). As shown in Table 3, each cognitive screening test had the highest AUC for detecting MCI in the group of Age > 70 years, Education ≤ 12 years. Comparison of independent ROC curves was carried out in different age and education level group for each cognitive screening test, respectively. Significant difference was found between the group of Age ≤ 70 years, Education ≤ 12 years and Age > 70 years, Education ≤ 12 years in the tests of C‐MACE, ACE‐III‐CV, and MMSE (*p* = .0012, .0108, .0026, .0412, respectively). The C‐MACE also had significantly higher AUCs in the group of Age > 70 years, Education > 12 years than in the group of Age > 70 years, Education ≤ 12 years (*p* = .0175, .0133, respectively). Though MoCA‐BC had the highest AUC in the group of Age > 70 years, Education ≤ 12 years, no significant difference was found in the four groups.

**TABLE 3:** Comparison of AUC among groups of different age and education level

AUC	Age ≤ 70 years, Education ≤ 12 years (MCI: *n* = 114, NC: *n* = 171)	Age ≤ 70 years, Education > 12 years (MCI: *n* = 21, NC: *n* = 116)	Age > 70 years, Education ≤ 12 years (MCI: *n* = 61, NC: *n* = 46)	Age > 70 years, Education > 12 years (MCI: *n* = 90, NC: *n* = 98)
C‐MACE	0.871	0.888	0.958^†^	0.888^‡^
ACE‐III‐CV	0.878	0.903	0.956^†^	0.915
MoCA‐BC	0.883	0.891	0.938	0.935
MMSE	0.719	0.777	0.816^†^	0.775

*Abbreviations*: AUC: area under the receiver operating characteristic curve; ACE‐III‐CV, Chinese version of Addenbrooke's Cognitive Examination III; MACE, Mini‐Addenbrooke's Cognitive Examination; MMSE, Mini‐Mental State Examination; MoCA‐BC, Chinese version of Montreal Cognitive Assessment‐Basic.

^†^ For significant difference of AUC between the group of Age ≤ 70 yeas, Education ≤ 12 years and Age > 70 years, Education ≤ 12 years (*p* < .05).

^‡^ For significant difference of AUC between the group of Age > 70 years, Education ≤ 12 years and Age > 70 years, Education > 12 years (*p* < .05).

## 4 DISCUSSION

This study developed a modified C‐MACE with five items derived from the ACE‐III‐CV. The measurement accuracy of each item was confirmed by the significant associations with standardized neuropsychological tests. Besides the internal consistency verified by Cronbach's coefficient, the C‐MACE has a good convergent validity with MoCA‐BC. No obvious ceiling and floor effects were found. Validation study verified that this C‐MACE had a satisfactory classification accuracy in detecting MCI from NC according to significantly higher AUC than MMSE and comparable to ACE‐III‐CV and MoCA‐BC.

In the process of developing C‐MACE, a statistically based method was used instead of clinical judgment or prior experience. As a result, with a total score of 38, this C‐MACE mainly includes two cognitive domains: 19 points for verbal episodic memory assessed by learning and recall a 7‐item name and address; 19 points for semantic memory assessed by verbal fluency of generating animals and naming of the pictures presented. This is consistent with the fact that episodic memory and semantic memory appeared to be substantially impaired in MCI and preclinical Alzheimer's disease (AD) (Mickes et al., 2007; Salmon, 2012). Previous studies showed that memory tests with free recall alone were more likely to generate high false positive and this shortage was overcome with the task of cued recall (Cullum et al., 1993; Kuslansky et al., 2002). Contrary to the original version of MACE, memory test in this C‐MACE contains both the items of free recall and recognition, and this may be good for the classification accuracy. On the other hand, the items of time orientation and clock drawing included in the original version of MACE were not contained in this C‐MACE according to the item analyses. However, this may not reduce the ability of C‐MACE in detecting MCI as deficits in time orientation and visuospatial ability usually appeared in the late stages of cognitive decline (O'keeffe et al., 2011; Salmon, 2012). ROC analyses were administered to explore the diagnostic accuracy of each brief cognitive screening test in this study. The optimal cutoff score for detecting MCI and corresponding sensitivity and specificity of these tests were determined by the maximum Youden index. The sensitivity could be improved at the cost of specificity by using a higher cutoff score, and vice versa (Soreide, 2009). As early detection of MCI may help decrease the risk of further cognitive decline (Langa & Levine, 2014), a higher cutoff score with good sensitivity and minimized false negative rate should be adopted in a population with high prevalence of cognitive impairment such as individuals with subjective memory complaint (Kielb et al., 2017). However, there is no modifying‐course therapy for MCI due to AD currently, and adverse psychological effects such as anxiety and depression may be incited once the prodromal phase of dementia was determined (Milne, 2010). In view of this, lower cutoff scores prefer specificity and reduce the false positive rate should be selected in community population with an unusually high prevalence of cognitive impairment. It should be noted that in our study, the optimal cutoff scores of MACE and ACE‐III‐CV for detecting MCI were both lower than the pre‐specified thresholds (82 and 88 for ACE‐III, 21 and 25 for MACE) (Beishon et al., 2019). We attribute this phenomenon to several reasons. First, a comprehensive neuropsychological criterion which showed higher diagnostic accuracy than conventional criteria was used for the diagnosis of MCI in this study (Bondi et al., 2014). Second, item such as episodic memory in ACE‐III‐CV was modified for reducing the influence of implicit memory. Third, the pre‐specified thresholds were generated from case–control studies and thus may lead to a risk of bias.

Diagnostic accuracy of cognitive assessment tools were highly correlated with the age and education level (Tavares‐Júnior et al., 2019). Hence, in this study, further comparisons were carried out to assess the classification accuracy of each cognitive screening test in different age and education level groups. As a result, the C‐MACE, ACE‐III‐CV, MoCA‐BC, and MMSE all had a highest AUC for detecting MCI in the group of Age > 70 years, Education ≤ 12 years. Participants in this group were relatively aged and had a lower education level, and the prevalence of cognitive impairment would be obviously higher. This is consistent with the fact that brief screening instruments can well detect cognitive impairment especially in populations with a higher prevalence of underlying dementia (Lin et al., 2013). In the participants of Education ≤ 12 years, the C‐MACE, ACE‐III‐CV, and MMSE had a significantly higher AUC for detecting MCI in the group of Age > 70 years than in the group of Age ≤ 70 years, though in the participants of Education > 12 years, there was no significantly difference between the groups of Age > 70 years and Age ≤ 70 years. This may attribute to the possibility that higher education level has the potential to reduce the ability of brief screening instruments in detecting MCI among the aged population. In the participants of Age > 70 years, the C‐MACE but not ACE‐III‐CV, MoCA, and MMSE had a significantly higher AUC for detecting MCI in the group of Education ≤ 12 years than in the group of Education > 12 years. This implied that, for identifying MCI in aged people, the very brief screening instruments do better in population with low education, though in aged population with high education, a relative comprehensive screening instrument may do better than the very brief ones for detecting MCI. It should be noted that the MoCA‐BC had no significant difference of AUC for detecting MCI in each age and education level group, and this feature may be more applicable for a population regardless of the age and educational level.

Although cognitive screening instruments were widely used, the underdiagnosis rate of cognitive impairment is still high and partly due to the pressure on screening time (Iliffe et al., 2009). Besides the relatively lengthy administration time, not all the items in cognitive screening instruments were deemed essential and contributed to the classification accuracy (Braekhus et al., 1992). In our study, five items derived from the ACE‐III‐CV formed C‐MACE. As a very brief cognitive screening instrument, neither specialist training nor specific test material is needed. However, this abbreviated version of ACE performed better than MMSE and was comparably accurate to the standard Chinese version of ACE‐III and MoCA‐BC in identifying MCI, especially in aged people with low education. Compared to the original version of MACE proposed by Hsieh et al. (2015), as the test of naming is more time saving than clock drawing, administration time of this C‐MACE is obviously shorter and this would be more applicable in a large population or busy clinical settings. In addition, progressive decline in cognition demonstrated by longitudinal cognitive assessments provides more evidence for MCI due to AD (Albert et al., 2011). Raters may easily obtain the previous scores of the C‐MACE for individuals assessed by ACE‐III‐CV in one's early stage and get a longitudinal cognitive comparation. However, as a brief screening tool, the C‐MACE will not provide a formal diagnosis, individuals screened as cognitive impairment usually need further comprehensive assessments.

Several limitations in this study should be noted. First, the C‐MACE was based on the administration of ACE‐III‐CV and not carried out as a unique test in this study. The standard administration procedure for this C‐MACE has not been determined. Second, the time interval between learning and recall in the item of 7‐item name and address came from ACE‐III‐CV, what would be an appropriate time interval in the C‐MACE need further investigation. Third, the number of participants diagnosed as MCI in the group of Age ≤ 70 years, Education > 12 years was relatively low and sampling bias may exist in this group.

In conclusion, this present study developed a modified C‐MACE with five items derived from ACE‐III‐CV. For detecting MCI, this C‐MACE showed satisfactory classification accuracy with significantly higher AUC than MMSE and comparable to ACE‐III‐CV and MoCA‐BC, especially in aged people with low education.

## CONFLICT OF INTEREST

The authors declare no conflict of interest.

## Data Availability

The data that support the findings of this study are available from the corresponding author upon reasonable request.
